# Air-Knife-Assisted Spray Coating of Organic Solar Cells

**DOI:** 10.1021/acsami.3c05306

**Published:** 2023-07-10

**Authors:** Emma L. K. Spooner, Elena J. Cassella, Joel A. Smith, Thomas E. Catley, Sam Burholt, David G. Lidzey

**Affiliations:** † Department of Physics and Astronomy, University of Sheffield, Hicks Building, Hounsfield Road, Sheffield S3 7RH, United Kingdom; ‡ Department of Electrical and Electronic Engineering, Photon Science Institute, University of Manchester, Oxford Road, Manchester M13 9PY, United Kingdom; § Department of Physics, Clarendon Laboratory, University of Oxford, Parks Road, Oxford OX1 3PU, United Kingdom; ∥ Diamond Light Source, Harwell Science and Innovation Campus, Didcot OX11 0DE, United Kingdom

**Keywords:** organic solar cells, organic photovoltaics, spray coating, energy materials, polymer

## Abstract

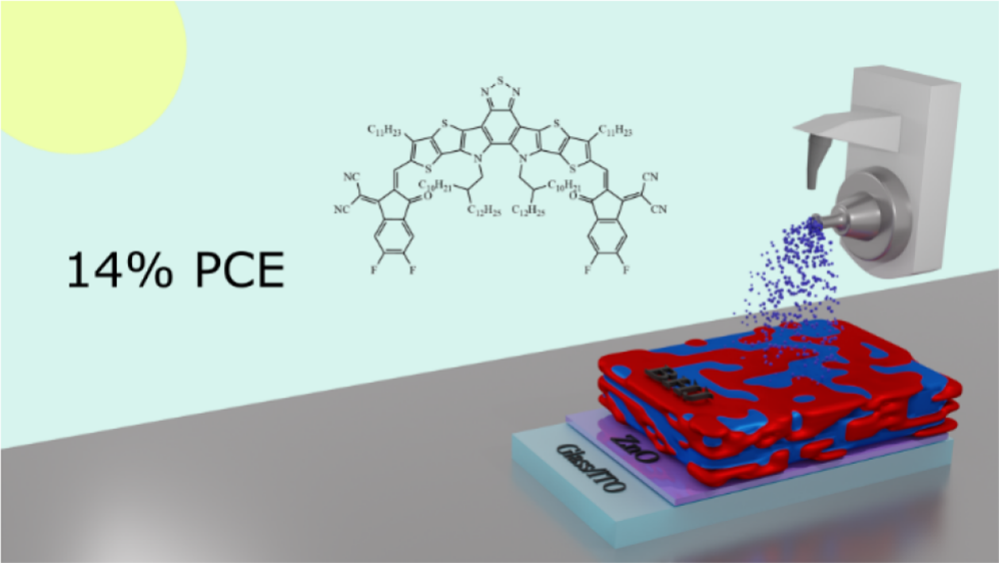

The power conversion efficiencies (PCEs) of organic solar cells (OSCs) have risen dramatically since the introduction of the “Y-series” of non-fullerene acceptors. However, the demonstration of rapid scalable deposition techniques to deposit such systems is rare. Here, for the first time, we demonstrate the deposition of a Y-series-based system using ultrasonic spray coating—a technique with the potential for significantly faster deposition speeds than most traditional meniscus-based methods. Through the use of an air-knife to rapidly remove the casting solvent, we can overcome film reticulation, allowing the drying dynamics to be controlled without the use of solvent additives, heating the substrate, or heating the casting solution. The air-knife also facilitates the use of a non-halogenated, low-toxicity solvent, resulting in industrially relevant, spray-coated PM6:DTY6 devices with PCEs of up to 14.1%. We also highlight the obstacles for scalable coating of Y-series-based solar cells, in particular the influence of slower drying times on blend morphology and crystallinity. This work demonstrates the compatibility of ultrasonic spray coating, and use of an air-knife, with high-speed, roll-to-roll OSC manufacturing techniques.

## 1 Introduction

The recent introduction of the “Y-series” non-fullerene acceptors (NFAs)^1^ has driven renewed interest in organic solar cells (OSCs), leading to record power conversion efficiencies (PCEs) approaching 19%.^2^ This class of NFAs is particularly notable for its enhanced near-infrared absorption (leading to record short circuit current values),^3^ high electron mobility (promoting long diffusion lengths),^4^ and low voltage losses.

While the efficiencies of such devices are now approaching those required for commercialization,^5^ the best-performing Y-series cells are still mainly fabricated using spin coating—a materially wasteful process that is incompatible with high-speed and high-volume roll-to-roll (R2R) manufacturing. Such devices are also typically fabricated using environmentally toxic, halogenated solvents such as chloroform. To propel the transition from “lab to fab”, it is necessary to develop scalable deposition technologies that both retain the PCEs of lab-scale devices and employ green solvent formulations.^6^

Although there has been success in fabricating such devices using R2R-compatible methods, progress has been mostly limited to the use of meniscus-based techniques such as blade^7−12^ and slot-die coating.^13−15^ The adoption of non-halogenated solvents in film deposition has also been complicated by the poor solubility and the tendency of many Y-series molecules to aggregate.^7^ Various methods have been used to overcome this, including deposition from hot inks (so-called “hot-casting”);^10,13,16^ the use of chemically modified acceptors such as DTY6,^7^ BTP-4F-12,^17^ BTP-BO-4Cl,^18^ and BTP-eC9;^12^ and the addition of solvent additives.^19^ Encouragingly, the efficiencies of blade-coated devices fabricated from non-halogenated solvents now approach 19%.^20^

Organic semiconductor devices can also be deposited via droplet-based techniques such as spray coating. Spray coating offers several key advantages over competing deposition processes—the most significant of which being that its noncontact nature permits materials and devices to be fabricated over nonplanar surfaces.^21^ Furthermore, spray coating has been estimated to have a far lower initial investment cost than techniques such as blade coating.^22^ Significantly, device fabrication by spray coating has been demonstrated at coating speeds as high as 12 m min^–1^—a rate that usually exceeds that of other common deposition techniques.^23^ Critically, enhancing the speed of high-throughput processing has been demonstrated to be a major contributing factor to enable the sustainable growth of solar manufacturing.^24^

In the spray coating process, an ink is formed into a mist and then carried to a substrate, where the sprayed droplets coalesce and dry, forming a thin film. The method of mist formation differs between techniques; for example, in ultrasonic spray coating, the ultrasonic vibration of a piezo-ceramic tip is used to break up the ink, which is then directed to the substrate using a gas jet. Previous work on spray coating of OSCs has included electrospray,^25^ ultrasonic,^26−29^ and airbrush spray coating.^30^ Both conventional and inverted devices and a range of PCBM-^31^ and NFA-based systems^32^ have previously been explored. Here, a record performance was achieved by Cheng et al. in 2020 who ultrasonically spray coated a PBDB-T-2Cl:IT-4F system, yielding PCEs in excess of 12%.^33^ To date, a Y-series-based system has not been fabricated via spray coating.

Recently, an alternative droplet-based aerosol “vibrating-mesh atomization” method has been developed by Yang et al.^16^ This technique was used to deposit both the charge-transporting layers and active layer of PTQ10:Y6-BO devices, creating fully printed devices with PCEs as high as 14.8%. However, we note that the slow 3 mm s^–1^ deposition speed of this technique, coupled with the requirement to heat the active layer solution to 80 °C, could lead to a process having relatively high manufacturing costs.

In this work, we use ultrasonic spray coating to fabricate OSC devices based on a blend of the polymer PM6 with the Y-series acceptor DTY6. Using the non-halogenated solvent *o*-xylene, devices with PCEs of up to 14.1% are obtained. Importantly, this process does not require solvent additives nor the necessity to apply any heating processes to either the casting solution or substrate, as both these protocols may be potentially difficult and costly to scale up to industrial levels.^34^ Instead, we control the wet film drying dynamics using an air-knife—a technique that has been demonstrated to be industrially scalable and has been used to “gas-quench” hybrid lead halide perovskite films in spray-coated cells^35^ and to assist drying in blade-coated OSCs.^12^

## 2 Results and Discussion

### 2.1 Methodology

**Figure 1:**
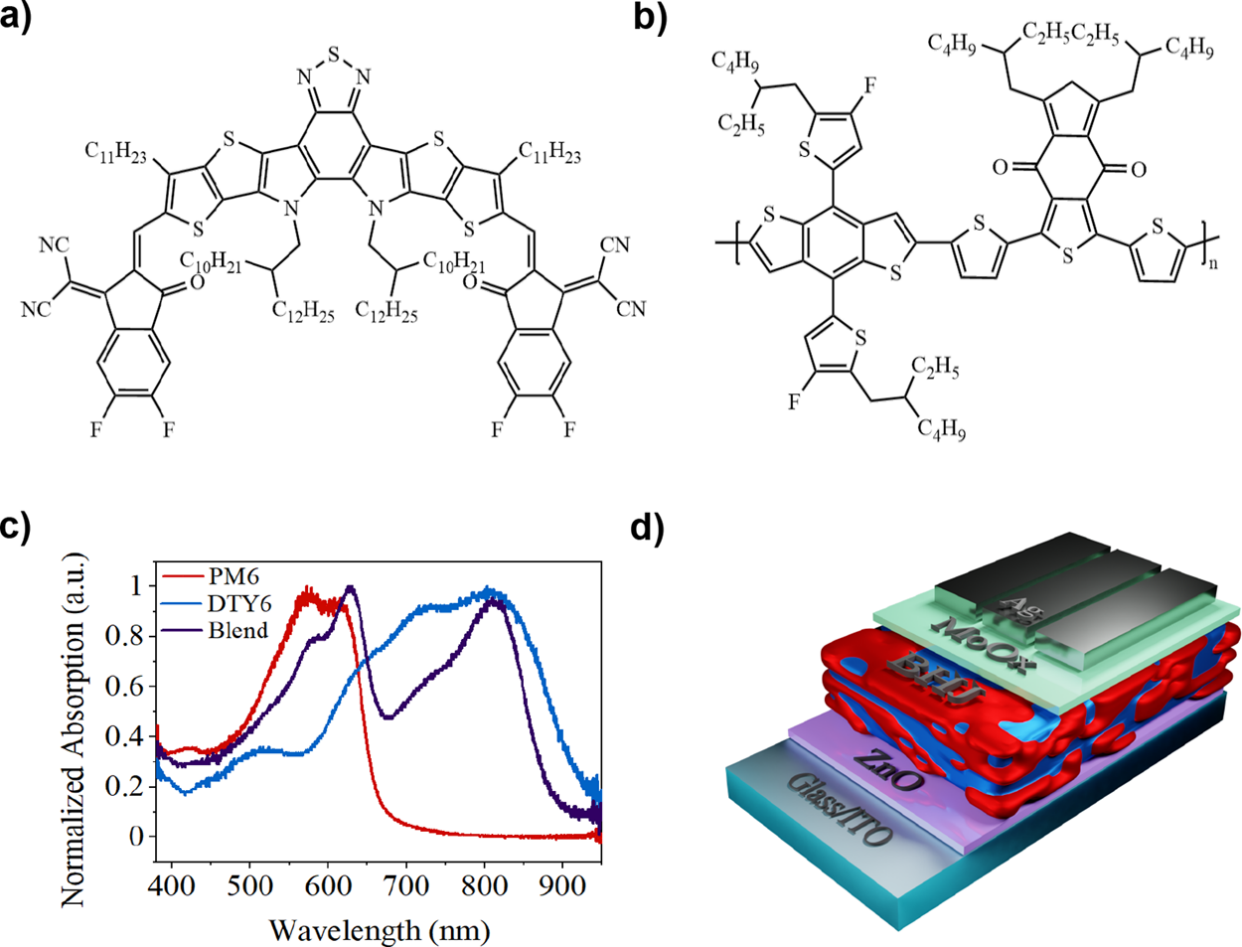
(a) Chemical structure of DTY6. (b) Chemical structure of PM6. (c) Thin film UV–vis absorbance of PM6, DTY6, and a 1:1.2 blend. (d) Schematic of the complete device architecture.

Devices were fabricated on an indium-doped tin oxide (ITO) cathode in an “inverted” architecture, with devices utilizing a spin-coated zinc oxide (ZnO) electron-transporting layer and thermally evaporated molybdenum oxide (MoO_3_) hole-transporting layer. The bulk heterojunction (BHJ) active layer was deposited by either spin coating or ultrasonic spray coating (referred to henceforth as “spray-coated devices”) from *o*-xylene. The BHJ consisted of a DTY6 (Figure 1a) acceptor and a PM6 (Figure 1b) donor in a 1:1.2 blend stoichiometry. The absorption of the blend components is shown in Figure 1c, and the complete device stack is shown in Figure 1d. Full details of all materials, fabrication techniques, and processes used are described in the Experimental Section.

### 2.2 Device Fabrication and Performance

**Figure 2:**
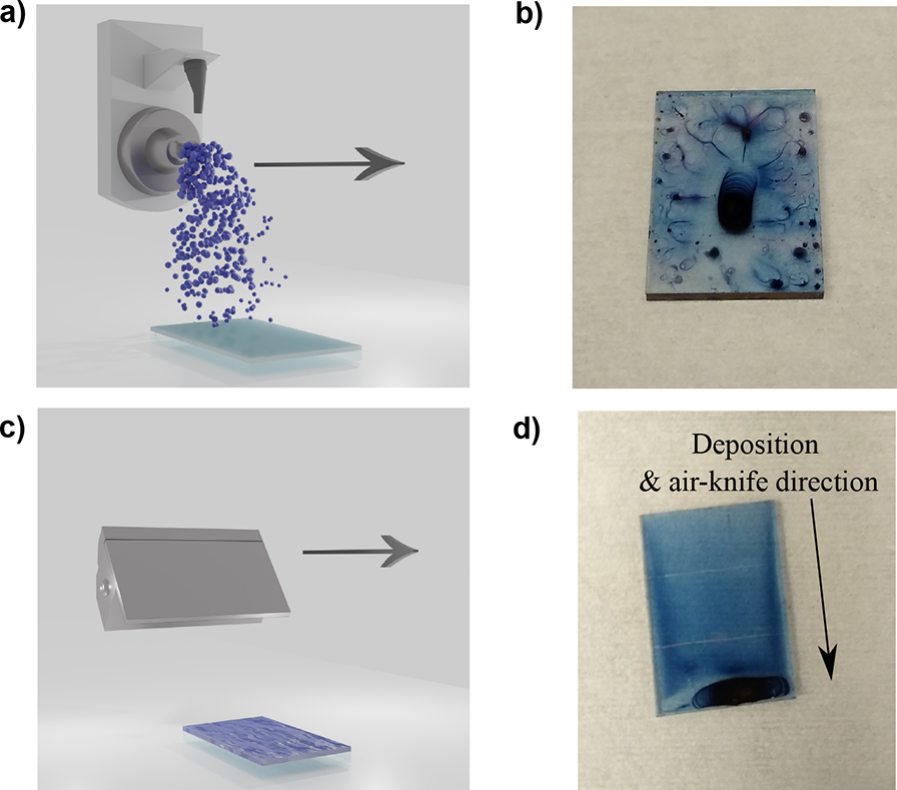
(a) Schematic of the spray coating head as it moves across the substrate. (b) PM6:DTY6 film coated using *o*-xylene, displaying significant reticulation. (c) Schematic of the air-knife moving across a spray-cast film. (d) PM6:DTY6 film coated using *o*-xylene, with application of an air-knife, showing superior coverage but with some material accumulation at the edge of the substrate.

Spray-coated devices were fabricated using a Sonotek Exactacoat system housed within a nitrogen-filled glovebox. During the spray coating process, the casting solution was fed into the spray head at a predefined flow rate, with a piezoelectric transducer used to generate ultrasonic vibration to break the solution into a series of uniform droplets. A gas jet was then used to guide the droplet mist toward the substrate surface. Following arrival at the surface, the droplets coalesced to form a continuous wet film. It was found that optimization of a series of parameters was necessary to deposit sufficient material to achieve the formation of a high-quality layer.^36^ These parameters included spray-coater head height, head speed, fluid flow rate, transducer driving power, substrate temperature, the pressure of the directing gas, and the nature of the casting solvent. In our optimized process, a motorized gantry was used to move the spray head linearly across the substrate surface at a speed of 20 mm s^–1^, with the substrate-to-head separation maintained at around 10 cm. The active layer solution was spray-cast in a single pass at a flow rate of 1.5 mL min^–1^ with the vibrating tip operating at a power of 1 W. This process is shown schematically in Figure 2a.

Although “Y-series” acceptors are typically deposited from chloroform, our initial experiments using PM6:Y6 active layers demonstrated that wet films spray-cast from chloroform did not undergo droplet coalescence (see Figure S1a,b). Here, the high vapor pressure of chloroform (boiling point 61 °C), coupled with the application of the directing gas, caused the droplets to dry upon contact with the substrate before they were able to coalesce.

Previous studies have shown that Y6 tends to aggregate unfavorably in high boiling point solvents,^7,10^ a finding that was reflected in our own attempts to coat PM6:Y6 in chlorobenzene/chloroform mixes, which yielded drastically reduced performance. Modification of the chemical structure of Y6 can however overcome this issue. For example, by creating the alkylated derivative DTY6, high-performance OSCs can be realized when cast from both low and high boiling point solvents,^7^ including the non-halogenated solvent *o*-xylene. *o*-Xylene has also been used to fabricate high-efficiency Y-series-based OSCs via blade coating,^10−12^ slot-die coating,^13^ and aerosol printing.^16^ In theory, *o*-xylene should therefore be a promising higher boiling point solvent for spray coating.

Inspired by this idea, we adapted our spray coating process to deposit a PM6:DTY6 blend from *o*-xylene. It was found, however, that spray-cast films underwent significant reticulation if the deposition substrate was either heated or maintained at room temperature (see Figure 2b). We note that solution de-wetting is not uncommon during spray-casting films due to the dilute nature of the casting ink, even though *o*-xylene has a low contact angle on ZnO (Figure S2b).^37^ This is less likely to occur in techniques such as spin coating and blade coating due to the presence of centrifugal and meniscus dragging forces, respectively. One strategy to overcome this issue is to deposit a moderately large amount of ink to form a continuous film; however, we found that the elevated drying times of high boiling point solvents such as *o*-xylene, together with surface tension effects, still resulted in reticulation.

To mitigate this effect, we have explored the use of an air-knife, which was passed over the wet film surface using a motorized gantry. Specifically, the air-knife was moved linearly across the substrate surface at a height of ∼2 cm, blowing a jet of N_2_ at 20 psi across the film surface shortly after the organic film has been spray-cast (see schematic in Figure 2c). We find that this process visibly encouraged the evaporation of the casting solvent and subsequently reduced the wet film drying time. Although the application of the air flow results in the loss of some of the spray-cast ink as it is “sheared off” (also resulting in an accumulation of material at the substrate edges), we find that the solution reticulation is largely overcome, with uniform films created having a high degree of surface coverage (see Figure 2d). Here, we believe that the air-knife both accelerates solvent evaporation and also spreads the wet film across the surface to some degree, with the combination of these effects leading to the formation of good quality films.

**Figure 3:**
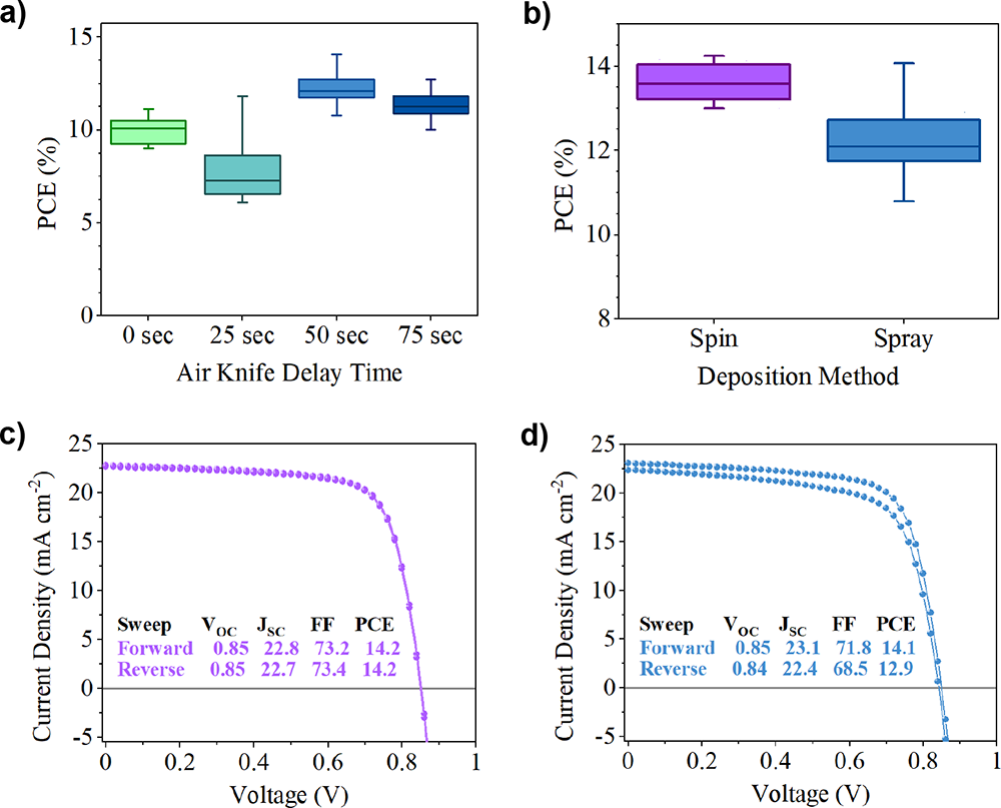
(a) Box plot of PCE for varying air-knife delay times. (b) Box plot of PCE for the optimized spray process compared to spin controls. (c) Champion spin *J*–*V* curve. (d) Champion spray *J*–*V* curve.

Our previous work on using an air-knife to control nucleation and crystallization of perovskite thin films indicated that the “delay time” between spray-casting the precursor solution and the application of the air-knife is a key optimization parameter that can be used to control the structure and morphology of the resultant perovskite films.^35^ We use a similar approach here, and—guided by device performance—we have optimized this delay time and find that device efficiency is maximized at a delay of 50 s (see Figure 3a).

Our measurements indicate that the air-knife delay time can be used to tune the thickness of the final film, with an extended delay time resulting in films having greater thickness (see Table S1). We suspect that this effect most likely results from increased solvent evaporation before the application of the gas jet, with the solution that is spread over the surface having increased concentration and viscosity. We find that this thickness increase is roughly correlated with an increase in ultraviolet–visible (UV–vis) absorption intensity, as shown in Figure S3b; however, we find that device metrics (Figure S3a and Table S1) do not follow the same trend.

In particular, performance is reduced for a 25 s delay time. We believe that this is in part due to large variations seen in device performance for lower delay times (0 and 25 s), as a result varied film quality. It is also likely that a combination of other factors influences device performance, including thickness variations, donor–acceptor phase separation, and aggregation of the components. The impact of air-knife delay time on active layer morphology is examined further in Section 2.4. Interestingly, we find that optimized devices based on spray-coated films generally require a thinner (∼90 nm) active layer than do those created by spin coating (∼140 nm). We therefore base our optimized spray-deposition process on a delay time of 50 s, which is used henceforward in all device fabrication experiments.

We first compare the performance of optimized spin- and spray-cast devices using a box plot in Figure 3b, with device metrics summarized in Table 1 and full metrics shown in Figure S4a. The current–voltage (*J*–*V*) curves for champion devices are also shown in Figure 3c,d. Encouragingly, we find that the champion performance of optimized spray- and spin-cast films is similar (see Table 1), with the highest efficiencies achieved being 14.1 and 14.2%, respectively. Significant photoluminescence quenching is also seen in both spin- and spray-coated blends, as shown in Figure S5, implying sufficiently high exciton quenching in both cases.^38^

**Table 1:** Device Metrics for Optimized Spin-Coated and Spray-Coated Devices^a^

deposition method	PCE (%)	FF (%)	*V*_OC_ (V)	*J*_SC_ (mA cm^–2^)
spin	13.6 ± 0.4 (**14.2**)	70.5 ± 1.5 (**73.4**)	0.85 ± 0.003 (**0.85**)	22.8 ± 0.70 (**24.0**)
spray	12.2 ± 0.8 (**14.1**)	68.0 ± 2.9 (**71.8**)	0.84 ± 0.01 (**0.85**)	21.4 ± 0.95 (**23.1**)

a Results are presented as an average of 10 cells ±1 standard deviation, with the champion cell efficiency shown in parenthesis. Forward and backward sweeps are treated separately but counted as 1 cell.

Despite the similar efficiency of champion devices prepared by spin and spray coating, we find a greater variation in performance for devices based on spray-cast films (see the histogram of device efficiency shown in Figure S4b) and a mean lower performance. It is evident that this enhanced variation in efficiency principally occurs from greater spread in both *V*_OC_ and *J*_SC_.

**Figure 4:**
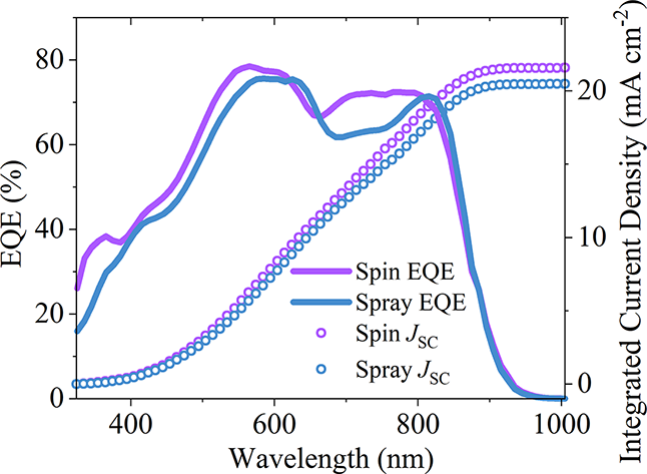
EQE curves of representative spin and spray devices, with integrated *J*_SC_ values shown.

To understand the origin of the difference in the efficiency of the spray- and spin-cast devices, we have characterized their external quantum efficiency (EQE), with the extracted integrated *J*_SC_ shown in Figure 4. We find that the integrated *J*_SC_ values determined from the EQE spectra match those determined using a solar simulator (see comparison detailed in Table S2), with any discrepancy between values being less than 10%.^39^ It is clear, however, that there are some differences between the EQE spectra for the spray- and spin-cast devices, with the EQE being relatively reduced around ∼700 nm in the spray-coated devices. If we compare this spectral region with the absorption of the PM6 donor and DTY6 acceptor (see Figure 1c), it appears that this reduction may be due to reduced photocurrent generation in the DTY6 component.

### 2.3 Optical Characterization

**Figure 5:**
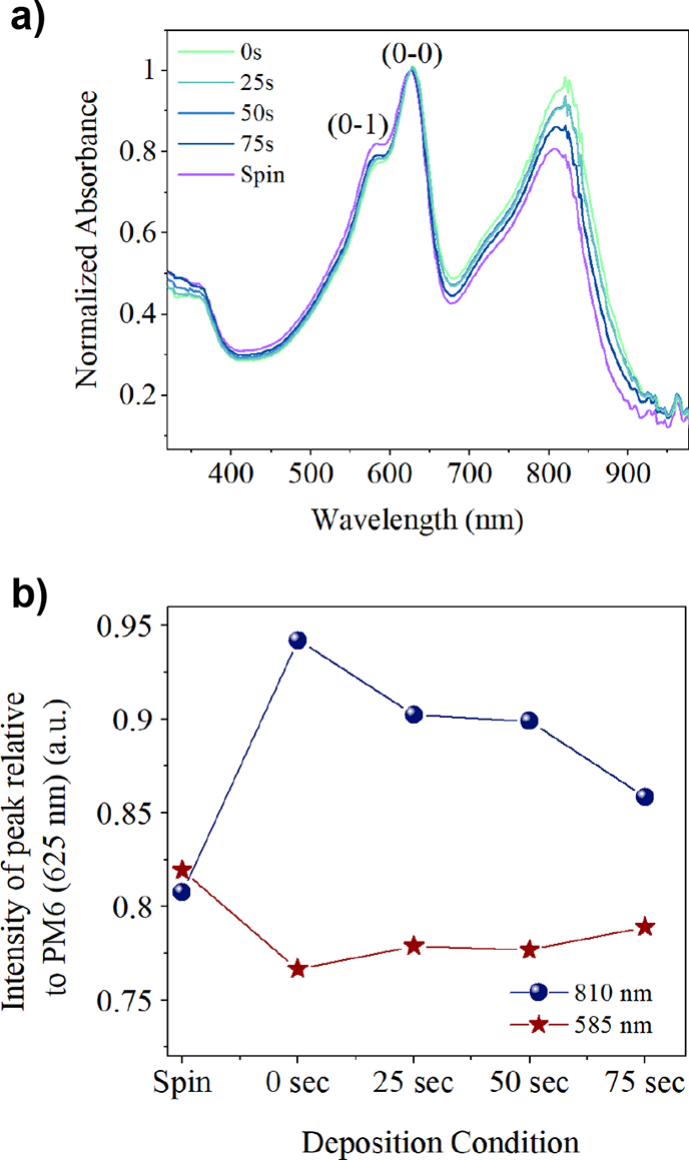
(a) UV–vis absorption spectrum for spin- and spray-coated films using varying air-knife delay times. Vibronic PM6 peaks marked. (b) Relative peak intensities with changing deposition conditions.

To explore this difference in EQE, we have measured the UV–vis absorption of control blend films. For completeness, data before normalization is shown in Figure S3b, where it is clear that all spray-coated films have reduced absorbance compared to the spin-coated control. Here, data is normalized to the main (0–0) PM6 peak at 625 nm, as shown in Figure 5a. It can be seen that there are small changes in the relative intensity of the PM6 (0–1) vibronic peak^32^ at ∼585 nm compared to the (0–0) peak, dependent on casting conditions. Specifically, we find that the (0–1) peak is most intense in blend films that have been spin-cast. We also observe small changes in the relative intensity of the (0–1) peak as a function of air-knife delay time; however, the statistical significance of these is small. We also find that the relative intensity of the peak of the DTY6 absorption (∼810 nm) is greatest in spray-cast films that are immediately air-knife quenched (i.e., delay time of 0 s). This absorption intensity is reduced as the delay time is increased and is smallest in the spin-cast films. To illustrate this, we plot the intensity of these peaks as a function of casting conditions in Figure 5b.

We believe that such changes in the intensity of the different peaks originate from different states of order, which are dependent on film casting conditions. Specifically, a reduction in the relative intensity of the (0–1) vibronic transition compared to the (0–0) electronic transition has been linked to stronger molecular aggregation and enhanced π–π stacking of polymer chains.^40^ As can be seen in Figure 5b, such increased aggregation of PM6 is observed in blend films that have been spray-cast films compared to those coated via spin coating. We suspect therefore that the deposition of the blend by spray coating offers more opportunity for molecular aggregation than occurs by spin coating in which the casting solvent is very rapidly removed. Such enhanced molecular packing can improve device efficiency;^41^ however, larger-scale aggregation and excess phase-separation, leading to the formation of domains or crystallites larger than the exciton diffusion length, is detrimental to device operation as a result of reduced exciton dissociation and charge generation.

Changes in the relative strength of the PM6:DTY6 absorption bands are also evident in Figure 5b but are more difficult to attribute to a single factor. Increased absorption of the DTY6 component relative to PM6 is seen in spray-cast films compared to spin-cast films, with this decreasing with increasing air-knife delay time. This may be due to a range of influences such as component aggregation^18^ and phase separation.^42^ We note that despite the lower EQE in the long-wavelength region for the spray-cast films, they actually have increased DTY6 absorption (relative to PM6) compared to those prepared by spin coating. This finding implies that the observed reduction in EQE is not simply caused by reduced DTY6 absorption but results from a relative reduction in charge generation efficiency.

To further understand the observed spectral differences and relate them to molecular order and morphology, we have used grazing-incidence wide-angle X-ray scattering (GIWAXS) to characterize blend films prepared under different casting conditions.

### 2.4 GIWAXS

**Figure 6:**
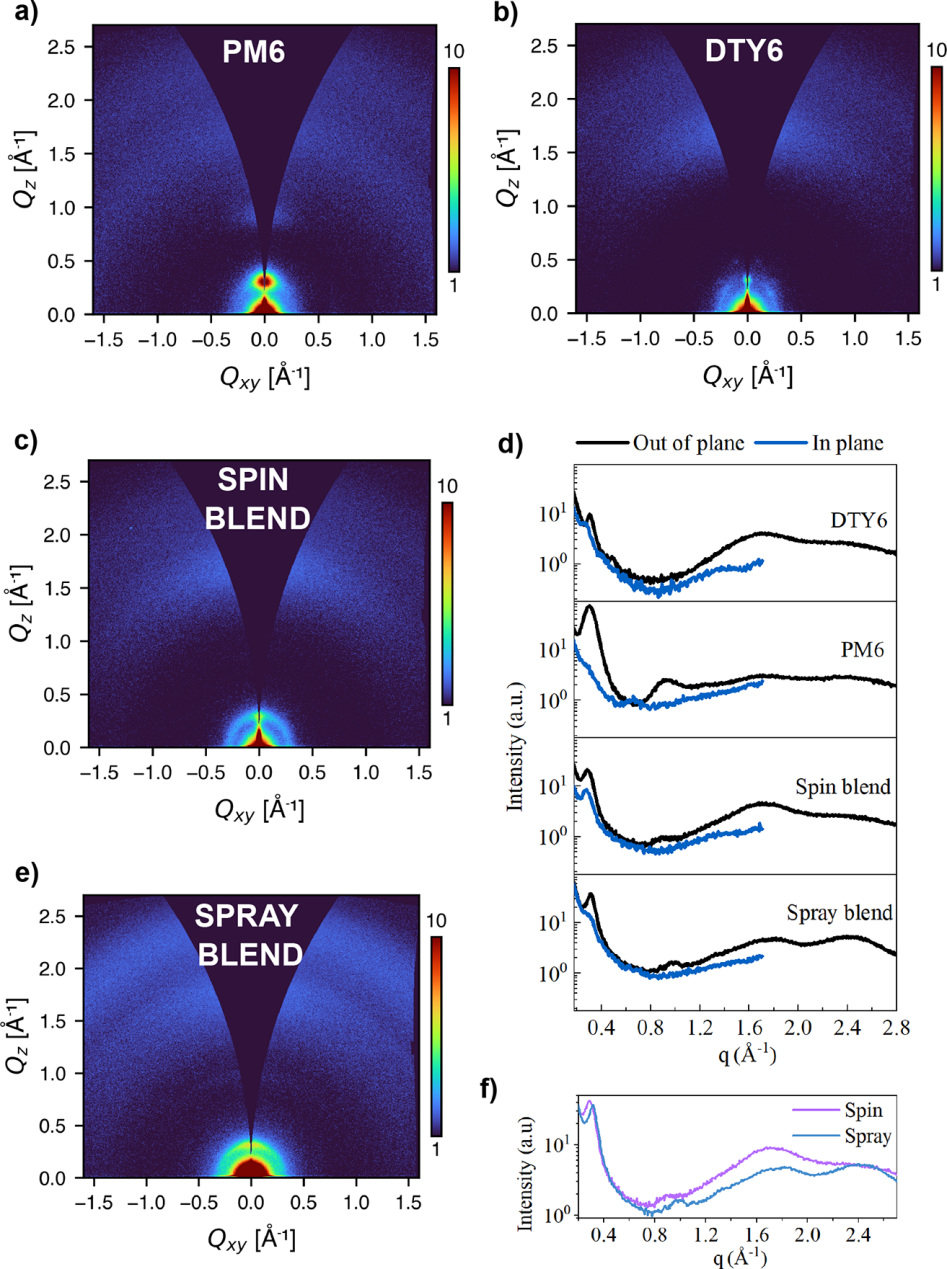
2D GIWAXS patterns for films on ZnO, shown as square root (intensity) to better clarify weaker features. Spin-coated (a) PM6, (b) DTY6, (c) PM6:DTY6 blend, (e) spray-coated PM6:DTY6 blend using optimum air-knife delay time. (d) 1D linecuts for blends and components. (f) 1D linecuts comparing out-of-plane intensity for spin- and spray-coated blends.

2D GIWAXS patterns for pure, spin-coated DTY6 and PM6 are shown in Figure 6a,b, respectively. Comparable patterns for PM6:DTY6 blend films coated via spin and optimized spray coating (delay time of 50 s) are shown in Figure 6c,e, respectively. Azimuthally integrated *q*-dependent 1D intensities for blends and components are shown in Figure 6d. The cut regions for the azimuthal integrations can be seen in Figure S6.

We first consider the pure components alone. Here, it can be seen that thin films of PM6 and DTY6 have several differences. From Figure 6a,d, it is apparent that PM6 undergoes prominent lamellar stacking in the out-of-plane direction (*q* ∼0.30 Å^–1^), with little stacking observed in the in-plane direction. A higher-order lamellar peak at *q* ∼0.92 Å^–1^ is also present in the out-of-plane direction. This implies preferential edge-on orientation, with a reasonably high level of order as suggested by the strong diffraction spot in Figure 6a. Weak π–π stacking peaks can be seen in the out-of-plane direction at *q* ∼1.7 Å^–1^ and *q* ∼2.4 Å^–1^. We suspect that the expected edge-on in-plane π–π stacking peaks at similar *q* values are beyond the limits of *q_xy_*. In contrast, DTY6 shows lamellar stacking in both directions (*q* ∼0.31 Å^–1^), with the more isotropic rings in Figure 6b suggesting a reduced degree of orientation and hence greater disorder than PM6. Strong π–π stacking can be seen at *q* ∼1.7 Å^–1^, suggesting that face-on orientation dominates, alongside weaker π–π stacking at *q* ∼2.4 Å^–1^. For clarity, an illustration of these orientations is shown in Figure S7.

The spin blend film shown in Figure 6c,d displays similar scattering to that of pure DTY6, with a more even distribution of lamellar stacking both in the in- and out-of-plane directions. The coexistence of face-on and edge-on orientations in PM6:DTY6 films has been reported in other work^7^ and is not thought to negatively impact device performance. Note that we cannot conclusively attribute the strong out-of-plane π–π stacking peak observed at *q* ∼1.7 Å^–1^ to either component due to a significant overlap of the peaks.

The spray blend film shown in Figure 6d,e displays some key differences to that of the spin blend. The crystal coherence length (CCL) can be calculated using Smilgies’ adaptation of the Scherrer equation,^43^ as shown in eq 1:
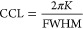
1Here, *K* is the Scherrer constant (taken to have a value of 1.0), with FWHM being the full width at half-maximum of the scattering peak. Using this equation, we determine that the in-plane lamellar peak of the spin blend at *q* ∼0.30 Å^–1^ is slightly broader in the spray-cast film, with the CCL reducing from 4.5 to 4.1 nm. The CCL provides an estimate of the lower limit of the crystalline domain size and here indicates a small reduction in the degree of lamellar crystallinity, likely that of DTY6.

For ease of comparison of the out-of-plane peaks, the 1D linecuts for the blends are displayed together in Figure 6f. From this figure, it is clear that the lamellar peaks (*q* ∼0.30 Å^–1^ and *q* ∼0.90 Å^–1^) are both shifted to higher *q* values in the spray-coated film, corresponding to decreased *d*-spacing. This is consistent with closer packing or aggregation of the edge-on aggregates of one or both of the components, with that at *q* ∼0.90 Å^–1^ likely due to PM6.

It is also clear that the spray-coated film has a relatively reduced intensity of the π–π stacking peak at *q* ∼1.7 Å^–1^ compared to the intensity of the peak at *q* ∼2.4 Å^–1^. This latter peak is more prominent in the PM6 component film shown in Figure 6a, and again likely results from π–π stacking, but at smaller *d*-spacing than that at *q* ∼1.7 Å^–1^. Here, the relatively enhanced intensity of the scattering peak at *q* ∼2.4 Å^–1^ in the spray-coated film compared to that at 1.7 Å^–1^ is suggestive of a film in which PM6 molecules are more closely packed—a finding consistent with the absorption spectra. These changes likely occur as the spray-cast film (before application of the air-knife) is expected to contain a relatively increased quantity of solvent, resulting in enhanced diffusion of the blend components compared to the spin-cast film in which solvent is rapidly removed. To explore such effects further, a range of optoelectronic measurements were carried out on devices containing spin- and spray-cast films.

### 2.5 Optoelectronic Measurements

**Figure 7:**
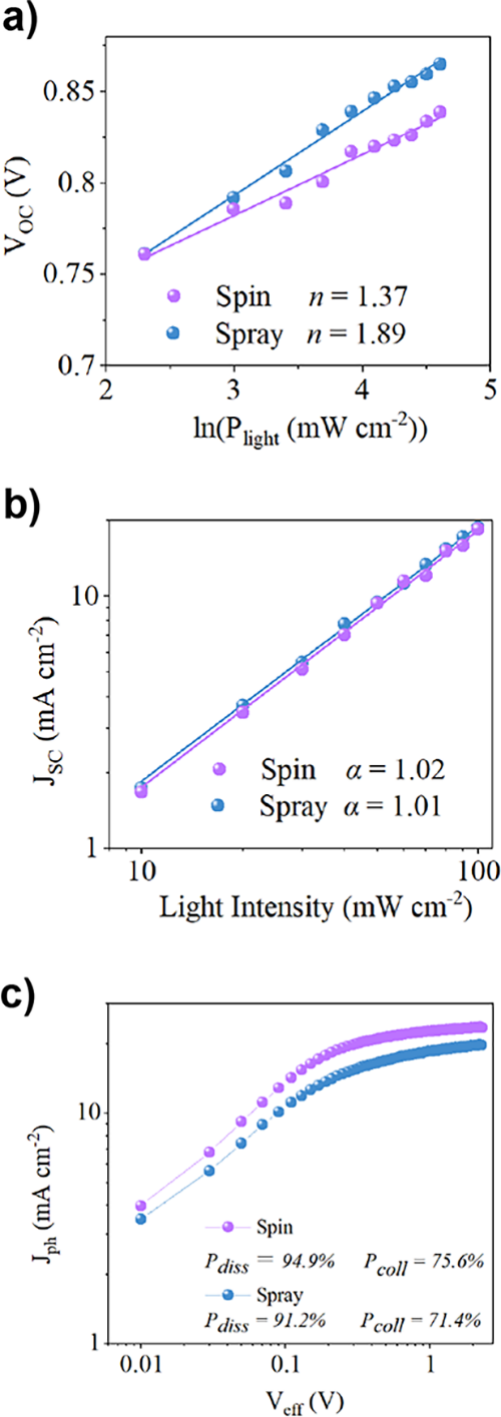
(a) Light-dependent *V*_OC_ measurements. (b) Light-dependent *J*_SC_ measurements. (c) *J*_ph_ measurements.

We have recorded light-dependent *J*–*V* curves for devices fabricated using the different deposition conditions, with Figure 7a plotting *V*_OC_ against the natural log of the light intensity [ln(*P*_light_)]. Here, the gradient of *V*_OC_ vs ln(*P*_light_) is expected to be , where *n*, *k*, *T*, and *q* are the ideality factor, Boltzmann’s constant, temperature, and elementary charge, respectively.^44^ It has been reported that as *n* approaches 2, trap-assisted recombination dominates over bimolecular recombination.^16^ Our data (shown in Table 2) indicates a value of *n* = 1.37 and 1.89 for the spin- and spray-cast devices, respectively, suggesting that spray-cast devices show significantly more trap-assisted recombination.

**Table 2:** Optoelectronic Properties Compared for the Two Deposition Conditions

deposition condition	*n*	∝	*P*_diss_	*P*_coll_
spin coated	1.37	1.02	94.9	75.6
spray coated	1.89	1.01	91.2	71.4

Figure 7b plots light-dependent *J*_SC_ measurements on a double-logarithmic scale. Here, we expect a *J*_SC_ ∝ *P*_light_^∝^ dependence, where ∝ is known as the “power factor”. Values of ∝ <1 indicate the enhanced presence of bimolecular recombination.^16^ Our measurements suggest values of ∝ of around unity for both types of devices (see Table 2), indicating similar and low levels of bimolecular recombination.

We also plot the photocurrent density (*J*_ph_) vs *V*_eff_ in Figure 7c. Here, *J*_ph_ is given by *J*_ph_ = *J*_L_ – *J*_D_ and was determined from the current recorded from dark (*J*_D_) and light (*J*_L_) *J*–*V* sweeps. This is plotted against *V*_eff_, where *V*_eff_ = *V*_0_ – *V*_appl_, with *V*_0_ being the voltage at which *J*_ph_ = 0 and *V*_appl_ being the applied voltage. Here, the photocurrent density is expected to reach a saturation value of *J*_sat_ at large *V*_eff_. This measurement is commonly used to determine the exciton dissociation efficiency (*P*_diss_ = ) at short circuit (*V*_appl_ = 0) and the exciton collection efficiency (*P*_coll_ = ) at the maximum power point.^41^ From our measurements (see Table 2), it can be seen that both the *P*_diss_ and *P*_coll_ values of the spray-coated devices are approximately 5% lower than those of spin-coated controls.

Our measurements suggest therefore that devices containing spray-cast films have slightly reduced levels of exciton dissociation and collection efficiency—a conclusion consistent with their reduced EQE (Figure 4). As the levels of bimolecular recombination are similar for the different deposition conditions, it seems likely that trap-assisted recombination drives this reduction in charge generation.

Typically, the closer packing and increased order of PM6 seen upon spray coating (e.g., Figure 5) would be associated with reduced structural defects and thus reduced trap-assisted recombination.^45^ Whether this is the case here however remains an open question; clearly, the reduced thickness of the active layer required to optimize the efficiency of the spray-cast device points to an increased density of traps that limits charge-carrier mobility. At present, such increased trapping in spray-cast films could occur in either the PM6 or DTY6 components, and we are currently unable to firmly distinguish between such possibilities. We note that it is possible that the spray process induces structural defects in the DTY6 domains of the BHJ. This would lead to electron trapping, resulting in increases in trap-assisted recombination^46^ and reduction in the DTY6 absorption region of the EQE upon spray coating. We note however that the order in the DTY6 component is difficult to probe using the techniques used here due to the lack of vibronic absorption peaks and overlap with PM6 GIWAXS features. Exploring the exact cause of the trap-assisted recombination is beyond the scope of this work but remains a topic of significant practical interest.

### 2.6 Larger-Scale Morphology

**Figure 8:**
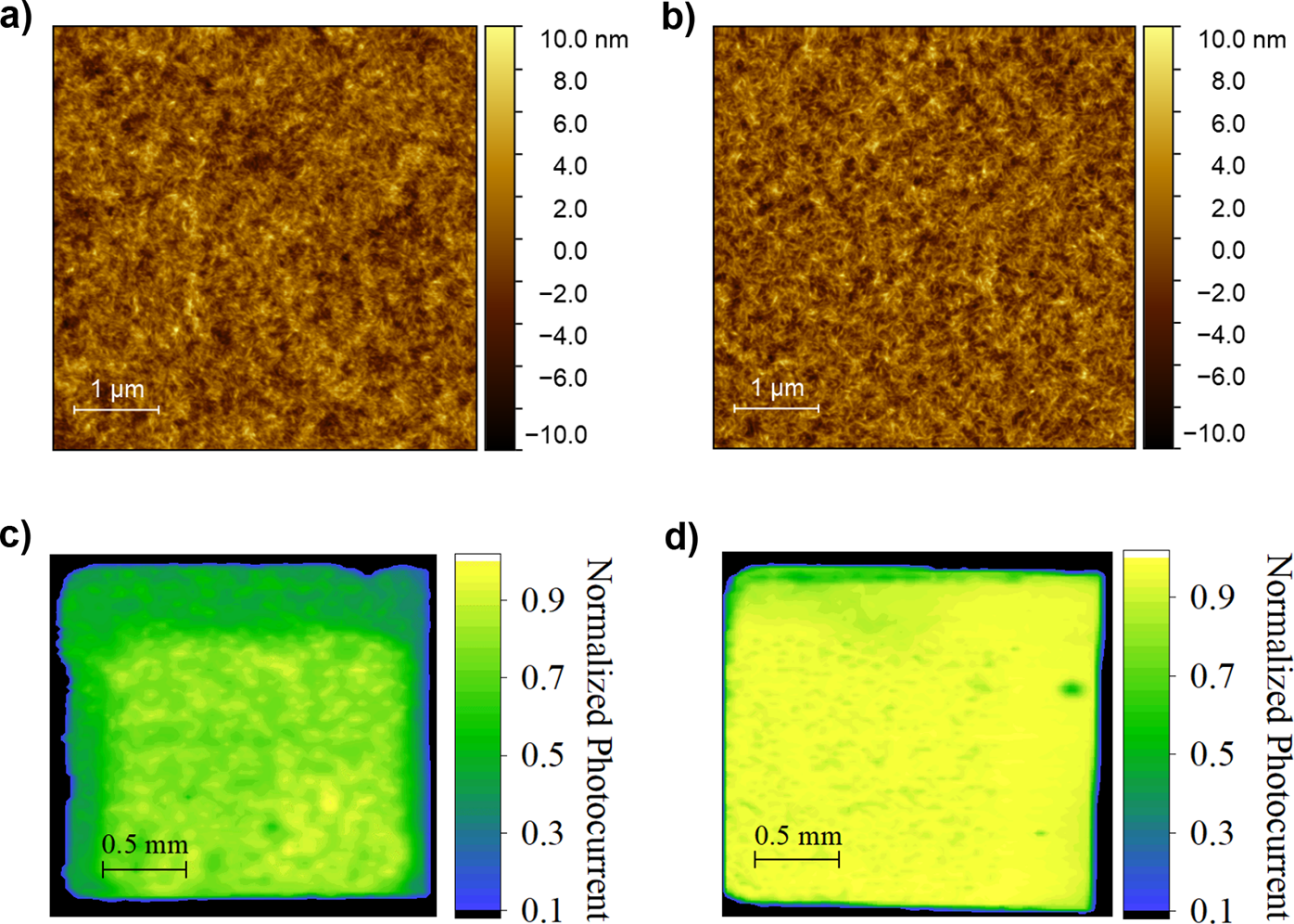
(a) AFM of the spin-coated film. (b) AFM of the optimized spray-coated film. (c) LBIC map of the spin-coated device. (d) LBIC map of the spray-coated device.

Finally, to explore whether larger-scale defects and morphological differences may also play a role, we have explored film uniformity over greater length scales. First, atomic force microscopy (AFM) was used to characterize films over a scan area of 5 μm × 5 μm. Here, representative images are shown in Figure 8a,b for optimized spin-cast and spray-coated films, respectively, with films spray-cast using different air-knife delay times shown in Figure S8. We find no apparent change in root-mean-square (RMS) roughness values (see Table S3), with film roughness in all cases being around 2.1 nm. However, a slightly more fibrillar network can be seen in the spray-cast film (Figure 8b). This is usually linked to superior performance, as a result of better domain connectivity and improved charge-carrier transport.^47^ We suspect that the fibrillar network formed here is a result of the “sit time” of the spray-cast film before application of the air-knife. Over the optimized 50 s delay time, the various components within the solution may undergo short-range pre-aggregation—a process that has been shown in other work to improve fibrillar formation via a “templating” effect.^48,49^ The tendency of PM6 to aggregate in solution^13^ and the greater PM6 aggregation seen in Figure 5 support this theory. Indeed, the formation of a network may allow spray-coated devices to achieve similar champion performance to the spin-coated samples at reduced film thickness. We note, however, that such structures may also be linked to the different levels of trap-assisted recombination between the films. Further investigation is required to establish the exact influence of fibrillar formation on performance.

To explore film homogeneity over a larger area, we used laser beam-induced current (LBIC) mapping to characterize photocurrent emission from spin- and spray-cast films over a scan area of 2 mm × 2 mm (see Figure 8c,d). While there is a surprising amount of non-uniformity in the spin-coated film, this does not severely impact device performance. Promisingly, the photocurrent generated across the spray-coated film appears slightly more uniform, although the statistical significance of this finding is unclear. We attribute the non-uniformity in the photocurrent seen in both devices to the high tendency for Y-series-based systems to phase separate over time.^50^

## 3 Conclusions

We have used an air-knife-assisted solvent extraction protocol to fabricate spray-coated OSCs with champion PCEs comparable to spin-coated control devices. Here, a nitrogen gas jet—applied by an air-knife moving linearly over the substrate surface—accelerates the evaporation of the casting solvent, preventing film shrinkage or aggregation effects that otherwise occur due to prolonged drying times. We note increased variation in spray-cast device performance and a small reduction in mean efficiency. We attribute reductions in performance to increases in trap-assisted recombination and a reduction in the efficiency of exciton collection and dissociation.

Relatively improved PM6 order and closer packing in spray-cast films is demonstrated from UV–vis absorption and GIWAXS measurements. We speculate therefore that spray-casting might introduce electron-trapping defects into DTY6 domains as a result of the increased drying time of the spray-cast films and potentially differing morphologies. At present, however, the exact origin of the increased trap-assisted recombination observed remains unclear. We also see a differing thickness–performance relationship for the spray-cast devices, which we believe may be due to the increases in trap-assisted recombination and/or differences in fibril formation, crystallization of components, or vertical segregation.

Despite this variation in performance, the champion spray PCE (14.1%) obtained was close to that of the spin-coated control (14.2%). Importantly, the spray-casting protocol developed does not require the use of additives to control solution rheology and is therefore ideal for low-cost, high-speed, R2R manufacturing. We believe that with modifications to the solvent system (e.g., using surfactants to improve wetting and droplet coalescence or a scalable solid solvent additive to tune molecular arrangement),^51^ this work marks an important first step toward developing a fully sprayed organic Y-series-based photovoltaic device.

## 4 Experimental Section

### 4.1 Materials

PM6 (poly[(2,6-(4,8-bis(5-(2-ethylhexyl-3-fluoro)thiophen-2-yl)-benzo[1,2-*b*:4,5-*b*′]dithiophene))-alt-(5,5-(1′,3′-di-2-thienyl-5′,7′-bis(2-ethylhexyl)benzo[1′,2′-*c*:4′,5′-*c*′]dithiophene-4,8-dione))]) was purchased from 1-Material. The 20 mm × 15 mm pre-patterned ITO glass (∼20 Ω/□) and DTY6 (2,2′-((2Z,2′Z)-((12,13-bis(2-decylteradecyl)-3,9-diundecyl-12,13-dihydro-[1,2,5]thiadiazolo[3,4-*e*]thieno[2″,3″:4′,5′]thieno[2′,3′:4,5]pyrrolo[3,2-*g*]thieno[2′,3′:4,5]thieno[3,2-*b*]indole-2,10-diyl)bis(methanylylidene))bis(5,6-difluoro-3-oxo-2,3-dihydro-1*H*-indene-2,1-diylidene))dimalononitrile) were purchased from Ossila. All solvents and remaining materials, including molybdenum(VI) oxide (99.97% trace metals basis), were purchased from Sigma-Aldrich, unless otherwise stated.

### 4.2 Substrate Preparation

ITO substrates (Ossila, S211) were cleaned via sonication in a dilute Hellmanex III solution, followed by dunk rinsing in boiling deionized (DI) water. Subsequent sonication in DI water, acetone, and isopropyl alcohol followed. Substrates were then dried via a N_2_ gun and subjected to UV-ozone treatment for 15 min prior to any layer deposition.

### 4.3 Electron Transport Layer

The ZnO precursor solution was prepared by dissolving ∼219 mg of zinc acetate dihydrate (99.99%) in 2 mL of 2-methoxyethanol (anhydrous, 99.8%), with the addition of 60.4 μL of ethanolamine (99.0%) before stirring overnight in ambient conditions. Prior to deposition, the solution was filtered through a polytetrafluoroethylene filter. The ZnO was then created via static spin coating at 4000 rpm to yield a layer of ∼35 nm. The film was patterned using a cotton swab dipped in methanol to expose the ITO and then annealed at 150 °C for 20 min before being transferred into a N_2_-filled glovebox.

### 4.4 Active Layer

Active layer solutions were made by dissolving 1:1.2 PM6:DTY6 in *o-*xylene (18 mg mL^–1^ for spin coating and 10 mg mL^–1^ for spray coating). Solutions were stirred at 80 °C overnight in a glovebox before being cooled to room temperature before use. Spin-coated films were formed via static deposition at 1500 rpm to form a film of ∼140 nm. Spray coating was performed using a Sonotek Exactacoat system using an Impact spray head. The piezoelectric tip was vibrated using a power of 1 W and the solution delivered at a flow rate of 1.5 mL min^–1^. The spray head was passed over the substrate at a speed of 20 mm s^–1^ at a tip-surface separation of ∼10 cm. After a short delay time, an automated gantry passed an air-knife (Meech A8 80 mm air-knife, RS components) held at a distance of ∼2 cm from the surface at a speed of 3 mm s^–1^, delivering N_2_ at a pressure of 20 psi. Optimized spray-coated films had a thickness of ∼90 nm. All active layer films were annealed at 80 °C for 10 min and then scratched using a razor blade to expose the underlying ITO contact. All coating and annealing were performed inside a N_2_-filled glovebox.

### 4.5 Hole Transport Layer and Cathode

A molybdenum(VI) oxide hole-transporting layer (10 nm) was thermally evaporated (Angstrom Engineering) from a RADAK (Luxel) source through a shadow mask at a constant rate of 0.1 Å s^–1^ and at a base pressure of at least 2.4 × 10^–6^ mbar. A silver contact (100 nm) was then deposited from a resistive source at a rate of 0.1–1.0 Å s^–1^ without breaking vacuum. The thickness of the evaporated film was monitored via a quartz crystal monitor.

### 4.6 Current–Voltage Measurements

All device *J–V* measurements were performed under ambient conditions against a matte black background using a Newport 92251A-1000 solar simulator whose power was adjusted to 100 mW cm^–2^ using an NREL-certified silicon reference cell. A Keithley 237 source-measure unit controlled by a custom-built code swept the devices at 0.2 V s^–1^ from 0 to 1.2 V. The area of the active device was defined by an aperture mask having an area of 2.5 mm^2^. Photocurrent density measurements were performed in the same way, but over a voltage range of −1.5 to 1.5 V, along with dark *J*–*V* sweeps.

### 4.7 EQE

EQE measurements were recorded using a Newport QuantX-300 quantum efficiency measurement system. The system is based on a 100 W xenon arc lamp focused through an Oriel Monochromator (CS130B), with light chopped at 25 Hz. Spectra were recorded over a 325–1000 nm wavelength range and referenced to a calibrated silicon cell.

### 4.8 Light-Intensity-Dependent Measurements

An Oriel LSH-7320 ABA LED solar simulator with adjustable output power between 0.1 and 1.1 suns was used to perform light-intensity-dependent *J*–*V* sweeps using the same sweep conditions as above.

### 4.9 LBIC Mapping

A 623 nm laser (Thorlabs, HRS015B) with a power of 1.2 mW and chopped at 500 Hz was focused onto the device electrode surface into a spot size of approximately 25 μm through a 10× objective lens. An XY stage (Zaber Technologies, X-LMS050A) was used to translate the sample in steps of 25 μm over the measurement area. The laser-generated photocurrent in the device was measured using a lock-in amplifier (Stanford Research Systems, SR830) and referenced to the chopped laser.

### 4.10 Profilometry

A Bruker DekTak XT surface profilometer was used to determine sample thickness. A razor blade was used to make scratches at multiple locations across the sample surface. A stylus (12.5 μm radius tip) was scanned with a force of 3 mg over a distance of 1000 μm at each location. Vision64 software was used to level the data at either side of the patterned region, before extracting a film thickness from the corresponding step height. Thickness measurements were averaged over multiple measurements from each sample.

### 4.11 AFM

AFM (Veeco Dimension 3100) samples were prepared using the same coating conditions specified above on ZnO-coated unpatterned ITO. Samples were measured in intermittent contact (tapping) mode with a NuNano Scout 350 cantilever (nominal spring constant 42 N m^–1^, resonant frequency 350 kHz). Each sample was scanned over three 5 × 5 μm^2^ areas with a resolution of 512 × 512 pixels. Gwyddion software was used to step line correct the images and extract the RMS roughness.

### 4.12 UV–Vis Absorption and Photoluminescence Measurements

Absorption samples were prepared using the coating conditions specified above onto quartz-coated glass substrates. Spectra were recorded using a FluoroMax 4 fluorometer (Horiba) using a Xe lamp. Photoluminescence measurements were performed on the same system using an excitation of 500 nm.

### 4.13 GIWAXS

GIWAXS measurements were performed on thin films prepared on ZnO-coated ITO glass. Samples were prepared using the same coating conditions as in devices. Measurements were taken on the DL-SAXS beamline at Diamond Light Source using a Xenocs Xeuss 3.0 system, with a liquid gallium MetalJet X-ray source. This source produced X-rays at an energy of 9.242 keV (λ = 1.341 Å) at an incidence angle of 0.15°. A Pilatus 1M detector at a sample-detector distance of ∼307 mm (calibrated using a silver behenate reference) was used to detect scattered X-rays. All GIWAXS measurements were taken under vacuum to reduce background scatter. Data was corrected, reduced, and reshaped using a custom Python code based on the PyFAI library.^52^ 2D images were given as square root (intensity) to better illustrate weaker features. Linecuts were produced via azimuthal integration in the range of −20° < χ < 20° for out of plane and 60° < χ < 90° for in plane.

## Supporting Information Available

Additional figures including photographs of films, further device data, and additional AFM images (PDF)
