# Supplementary material for: Air-Knife-Assisted Spray Coating of Organic Solar Cells
Source: ACS Appl Mater Interfaces. 2023 Jul 10;15(33):39625–35. doi: 10.1021/acsami.3c05306 (PMC10450690; doi:10.1021/acsami.3c05306)
Supplement: Supplementary file 1 — am3c05306_si_001.pdf [file am3c05306_si_001.pdf]

Supporting Information

Air-Knife Assisted Spray Coating of Organic Solar Cells

Emma L. K. Spooner,[*,‡,†] Elena J. Cassella,[‡] Joel A. Smith,[§] Thomas E. Catley, Sam Burholt[#] and David G. Lidzey[*]*

Department of Physics and Astronomy, University of Sheffield, Hicks Building, Hounsfield Road, S3 7RH, United Kingdom.

[†]Photon Science Institute, Department of Electrical and Electronic Engineering, University of Manchester, Oxford Road, M13 9PY, United Kingdom.

[§] Department of Physics, University of Oxford, Clarendon Laboratory, Parks Road, Oxford, OX1 3PU, United Kingdom.

[#] Diamond Light Source, Harwell Science and Innovation Campus, Didcot, OX11 0DE, United Kingdom.

[‡] E.L.K.S and E.J.C. contributed equally to this work

*Corresponding authors. D. G. Lidzey: d.g.lidzey@sheffield.ac.uk. E. L. K. Spooner: emma.spooner@manchester.ac.uk.

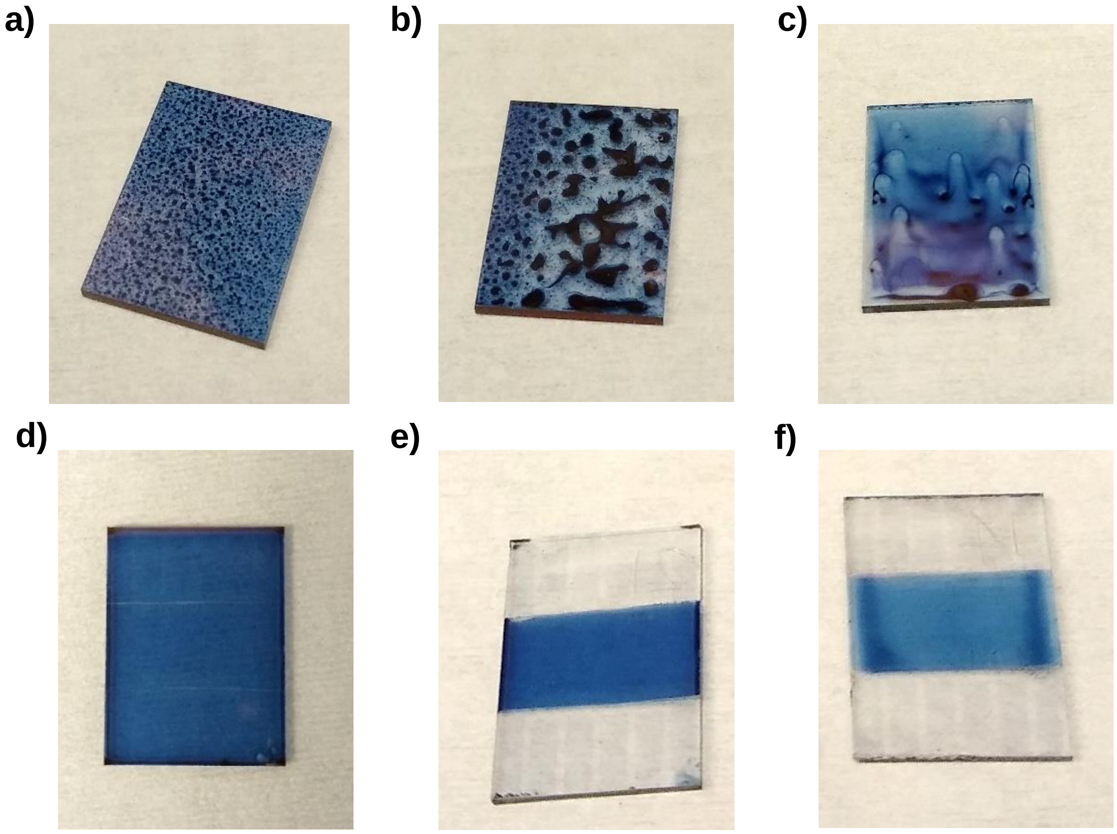

Figure S1: a) & **b)** PM6:Y6 films coated from chloroform, showing poor coalescence. **c)** PM6:DTY6 film coated from o-Xylene, with insufficient solution deposited leading to coalescence issues. A PM6:DTY6 film spin coated from *o*-Xylene **d)** before and **e)** after electrode patterning**. f)** Optimised spray coated film after patterning.

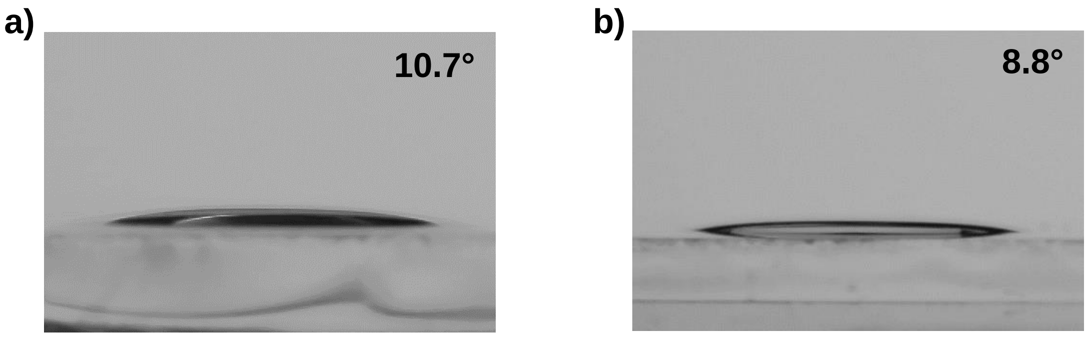

Figure S2: Contact angles of **a)** chloroform on ZnO, **b)** *o*-Xylene on ZnO.

**Deposition Method**	**Thickness of Film (nm)**	**J_{SC} (mA cm^{-2})**
Optimised spin coated	139 ± 5.4	22.8 ± 0.7
Spray coated- 0 second air-knife delay	70 ± 2.1	20.1 ± 0.8
Spray coated- 25 second air-knife delay	84 ± 1.7	19.1 ± 0.4
Spray coated- 50 second air-knife delay	90 ± 4.4	21.4 ± 1.0
Spray coated- 75 second air-knife delay	114 ± 3.3	21.3 ± 0.5

Table S1: Thin film thickness and corresponding J_{SC} values for each deposition condition.

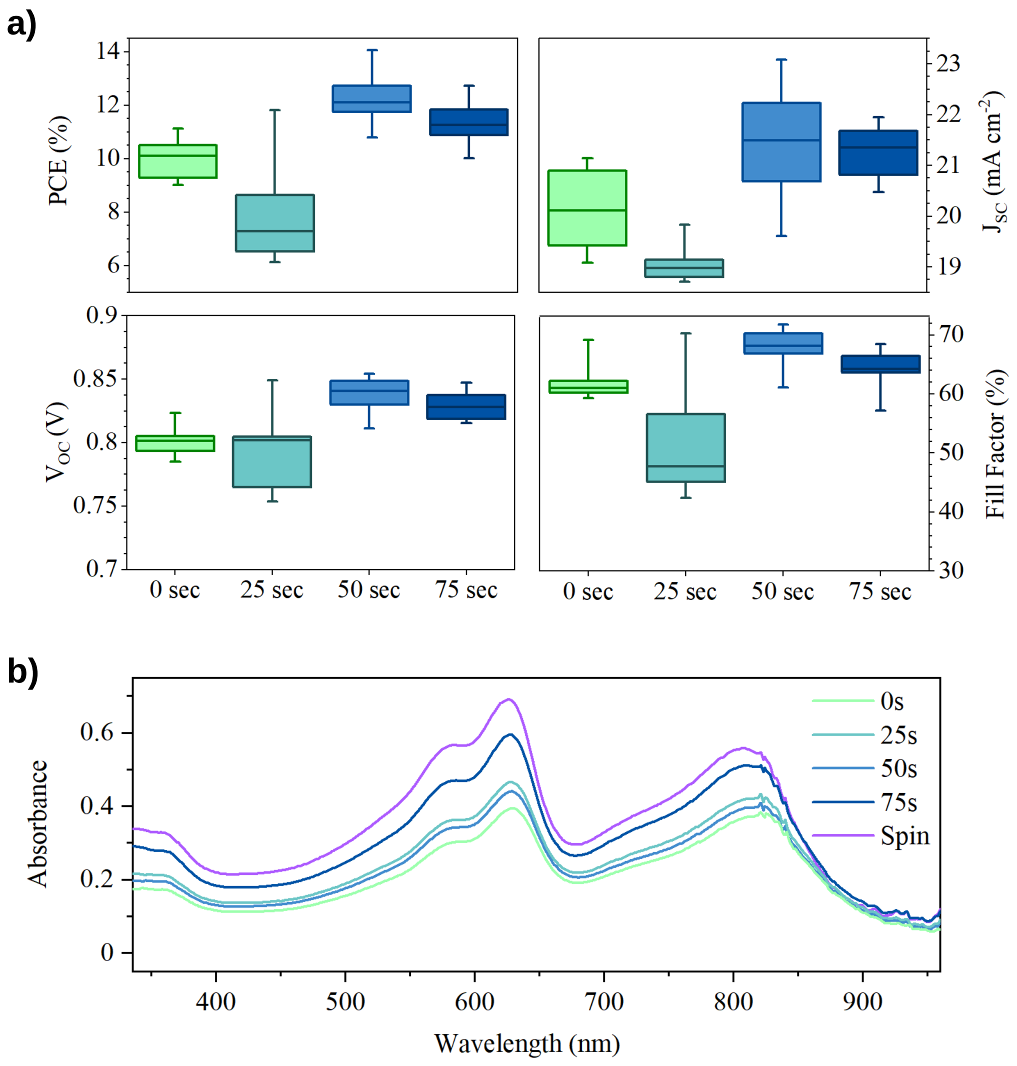

Figure S3: a) Box plots of device metrics for spray coated PM6:DTY6 devices with varying air-knife delay time. **b)** Absorbance of PM6:DTY6 thin films with varying air-knife delay time, without normalization.

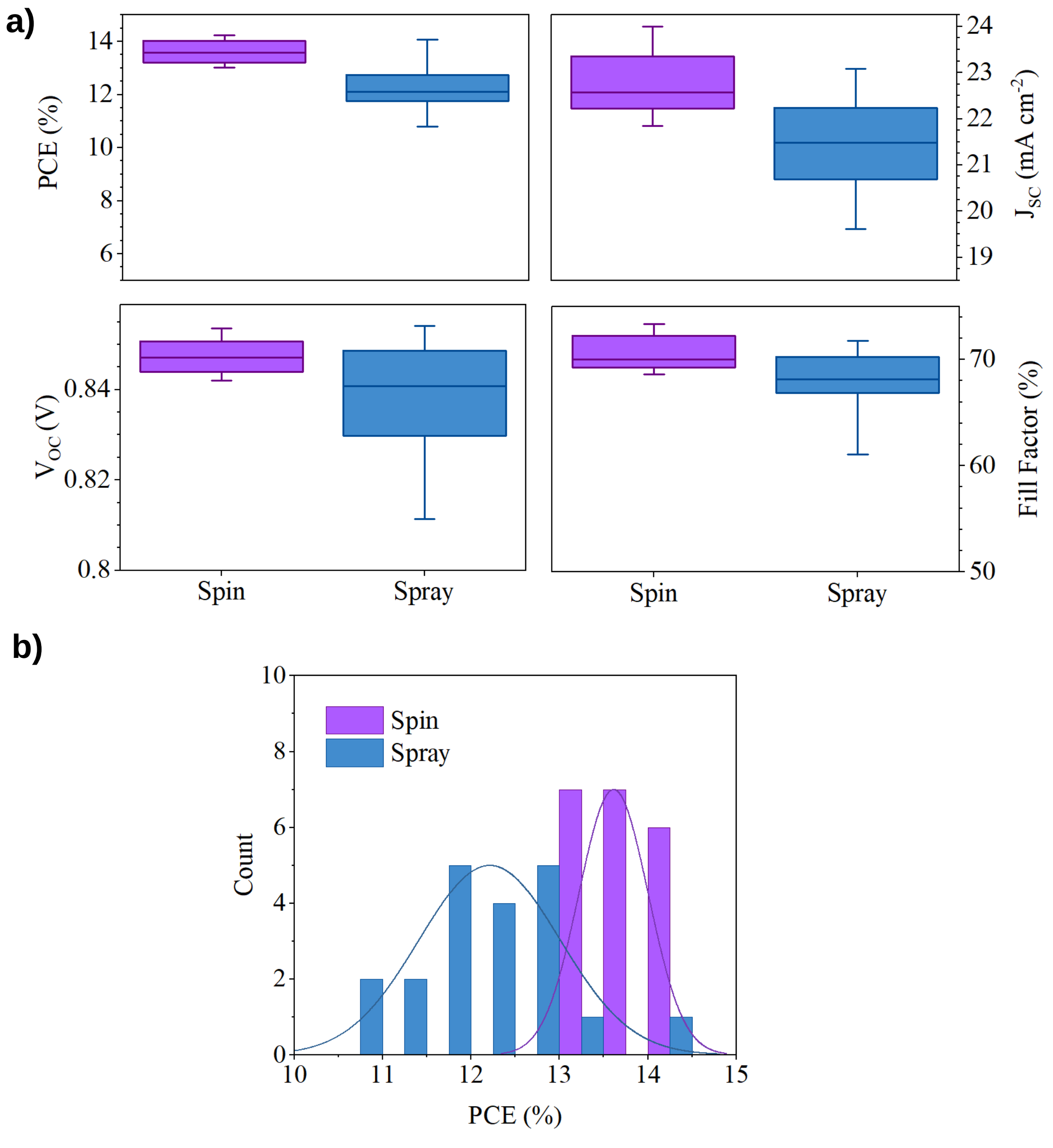

Figure S4: a) Box plots of device metrics for spin and spray coated (50 second air-knife delay time) PM6:DTY6 devices. **b)** Histogram of spin and spray device efficiencies, with 10 separate devices shown for each, but forward and reverse sweeps treated separately.

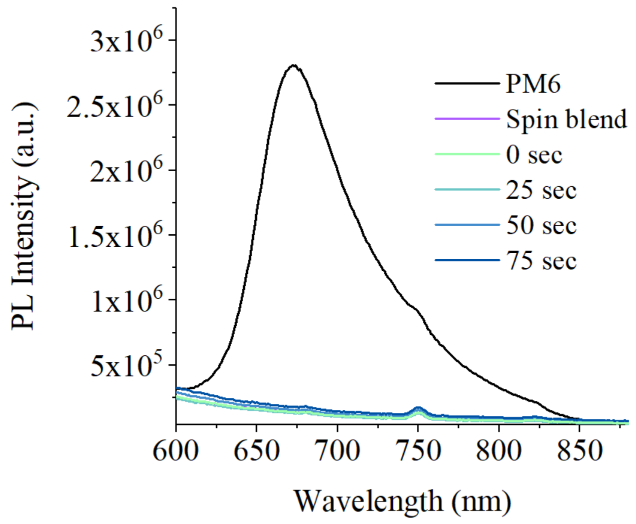

Figure S5: Photoluminescence intensity for spin and spray coating films. All films excited at 500 nm. The feature at 750 nm is attributed to an artifact of the diffraction grating.

Table S2: J_{SC} values for representative devices, compared to those achieved via integration of the EQE spectra.

Deposition Method	Measured J_{SC-JV} (mA cm^{-2})	Calculated J_{SC-EQE} (mA cm^{-2})	Discrepency (%)
Spin	23.9	21.6	9.6
Spray	20.8	20.4	1.9

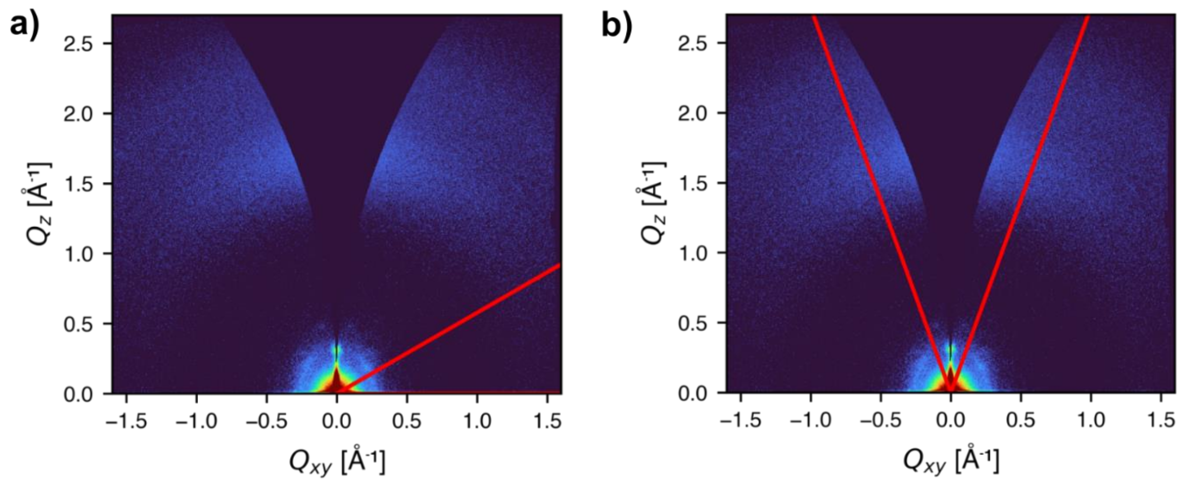

Figure S6: Cut regions for the azimuthal integrations shown Figure 6d and 6f, for **a)** in plane and **b)** out of plane regions.

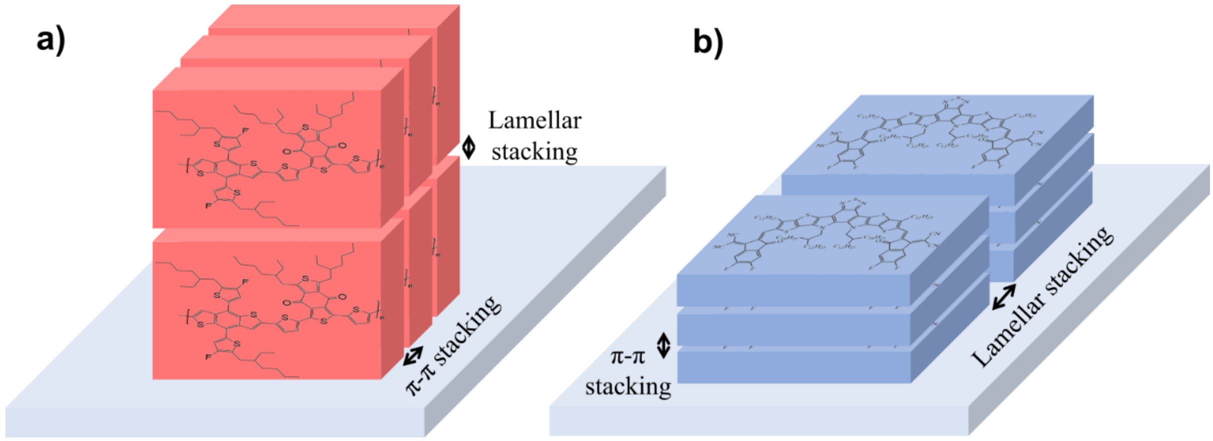

Figure S7: Illustration of stacking and relative orientations inferred from GIWAXS for **a)** PM6 (edge-on) and **b)** DTY6 (face-on).

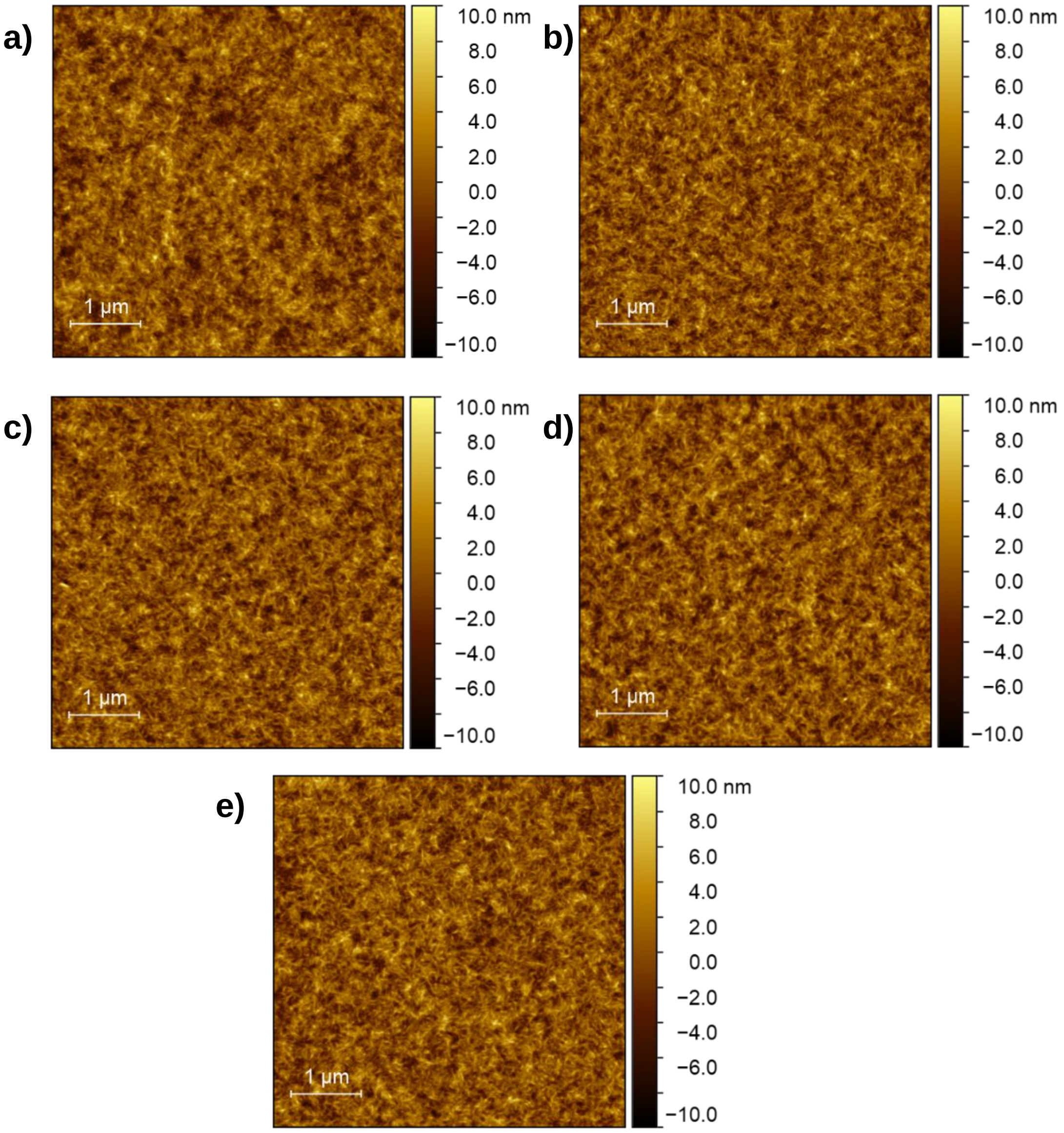

Figure S8: AFM images for **a)** Spin coated control. Spray coated films follow at **b)** 0 seconds air-knife delay time, **c)** 25 seconds, **d)** 50 seconds, **e)** 75 seconds.

Table S3: Root mean square (RMS) roughness values for each deposition condition calculated from the AFM data. Values given as an average of 3 different measurements across one film, with the error ± 1 standard deviation.

**Deposition Method**	**RMS Roughness (nm)**
Spin coated	2.11 ± 0.10
Spray coated- 0 second air-knife delay	2.06 ± 0.01
Spray coated- 25 second air-knife delay	2.08 ± 0.06
Spray coated- 50 second air-knife delay	2.09 ± 0.02
Spray coated- 75 second air-knife delay	2.06 ± 0.03
